# Distinct role of ERp57 and ERdj5 as a disulfide isomerase and reductase during ER protein folding

**DOI:** 10.1242/jcs.260656

**Published:** 2023-01-19

**Authors:** Philip John Robinson, Marie Anne Pringle, Bethany Fleming, Neil John Bulleid

**Affiliations:** School of Molecular Biosciences, College of Medical Veterinary and Life Sciences, Davidson Building, University of Glasgow, Glasgow G12 8QQ, UK

**Keywords:** Disulfide formation, Endoplasmic reticulum, Protein disulfide isomerase, Protein folding, Protein secretion

## Abstract

Proteins entering the secretory pathway need to attain native disulfide pairings to fold correctly. For proteins with complex disulfides, this process requires the reduction and isomerisation of non-native disulfides. Two key members of the protein disulfide isomerase (PDI) family, ERp57 and ERdj5 (also known as PDIA3 and DNAJC10, respectively), are thought to be required for correct disulfide formation but it is unknown whether they act as a reductase, an isomerase or both. In addition, it is unclear how reducing equivalents are channelled through PDI family members to substrate proteins. Here, we show that neither enzyme is required for disulfide formation, but ERp57 is required for isomerisation of non-native disulfides within glycoproteins. In addition, alternative PDIs compensate for the absence of ERp57 to isomerise glycoprotein disulfides, but only in the presence of a robust reductive pathway. ERdj5 is required for this alternative pathway to function efficiently indicating its role as a reductase. Our results define the essential cellular functions of two PDIs, highlighting a distinction between formation, reduction and isomerisation of disulfide bonds.

## Teaser

**Summary:** Two members of the PDI family, ERp57 and ERdj5, assist secretory protein folding by correcting non-native disulfide bonds. Their contrasting dependency on a reducing pathway reflects their different roles as isomerase and reductase.

## INTRODUCTION

The endoplasmic reticulum (ER) is the main cellular compartment where the folding of secretory proteins takes place (Ellgaard et al., 2016). An important part of the protein folding process is the formation of native disulfide bonds. This not only involves the coupling of cysteine residues to form disulfides but also the isomerisation of aberrant non-native disulfide bonds (Ellgaard et al., 2018). These reactions are catalysed by the protein disulfide isomerases (PDIs), a family of over 20 oxidoreductase enzymes resident in the ER, which can introduce or remove disulfides through thiol-disulfide exchange mechanisms (Okumura et al., 2015; Robinson and Bulleid, 2020). The redox potential of the oxidoreductase active site relative to the substrate largely determines whether the enzyme will reduce or oxidise disulfides (Hudson et al., 2015; Jensen et al., 2009), but kinetic factors, such as substrate binding and steric interactions, also influence how the oxidoreductases function (Ellgaard et al., 2018; Hudson et al., 2015). In general, PDIs with a relatively high reduction potential will act as oxidases, whereas those with relatively low reduction potentials will act as reductases. To catalyse isomerisation of disulfides, therefore, requires an active site that has a balance of reductase and oxidase potential.

**Fig. 1.**
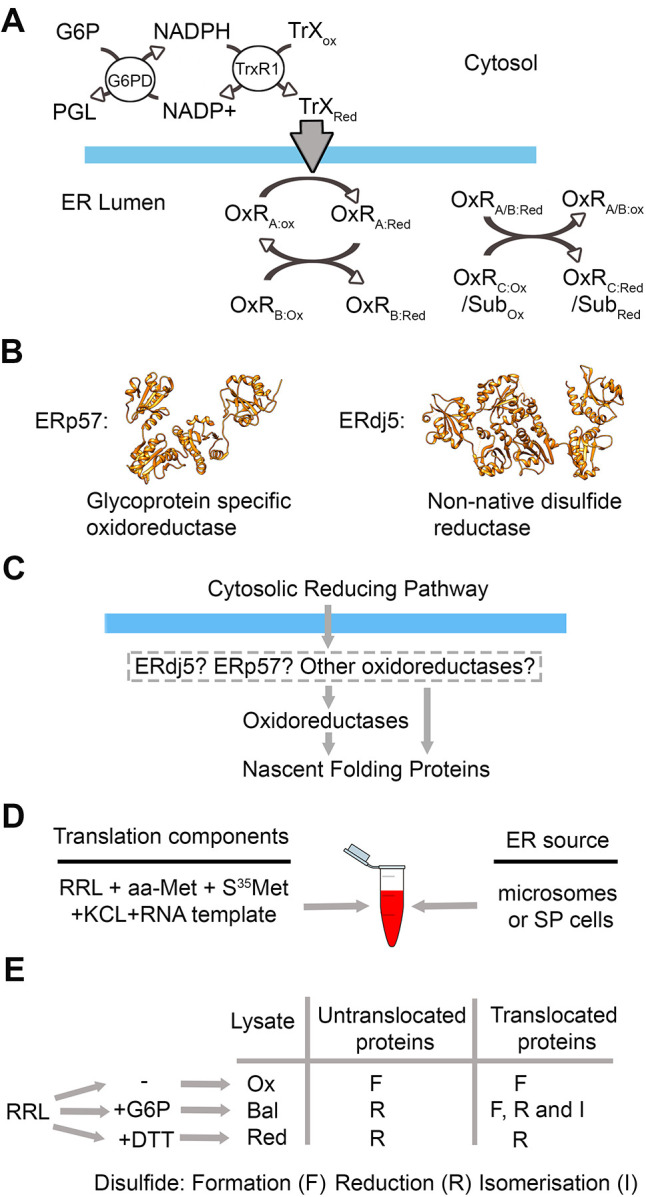
**A translation system to assay the role of ERp57 and ERdj5 in the ER reducing pathway.** (A) The cytosolic reducing pathway, involving glucose 6-phosphate dehydrogenase (G6PD) and thioredoxin reductase (TrxR1), is the source of reducing equivalents required to reduce ER localised oxidoreductases (OxR). OxR_Red_ can either reduce other oxidoreductases (OxR_Ox_ to OxR_Red_) or substrates (Sub_Ox_ to Sub_Red_). PGL, phosphoglucolactone; TrX, thioredoxin. (B) Ribbon structures of ERp57 (PDB: 3F8U) and ERdj5 (PDB: 3APO), two oxidoreductases that are involved in disulfide reduction or isomerisation during protein folding. (C) The possible routes by which reducing equivalents are transferred from the cytosol to nascent folding proteins in the ER. (D) Components of a typical translation reaction – rabbit reticulocyte lysate (RRL), amino acids minus methionine (aa-Met), S^35^-labelled methionine (S^35^ Met), potassium chloride (KCL) and RNA template. Microsomes or semi-permeabilised (SP) cells are added as an ER source. (E) The redox status of untreated rabbit reticulocyte lysate (RRL) is oxidised (ox) and is altered by adding G6P to form redox-balanced lysate (Bal) or DTT to form reducing lysate (Red). These supplements impact disulfide formation, reduction and isomerisation as shown in the table.

The reduction of a disulfide results in the catalytic cysteine residues becoming oxidised. To carry out further rounds of reduction, an ER-reductive pathway is required to recycle the oxidoreductase back to its reduced form. Previously, we have shown that a major source of reducing equivalents required for the recycling of oxidoreductases originate in the cytosol (Poet et al., 2017) (Fig. 1A). The reducing equivalents enter the ER via an unknown membrane protein (Cao et al., 2020) and are potentially transferred through the network of oxidoreductases via thiol-disulfide exchange (Oka et al., 2015). The two oxidoreductases that are known to reduce or isomerise non-native disulfide bonds are ERp57 (also known as PDIA3; Jessop et al., 2007) and ERdj5 (also known as DNAJC10; Oka et al., 2013) (Fig. 1B). ERp57 can efficiently catalyse the formation, reduction and isomerisation of disulfide bonds *in vitro*, which indicates that it has a balanced reduction potential and might catalyse diverse reactions *in vivo* (Frickel et al., 2004). As an enzyme of the calnexin cycle, ERp57 acts primarily on N-linked glycoproteins (Kozlov and Gehring, 2020). In contrast, the substrate specificity of ERdj5 is thought to be due to its interaction via its J-domain with the ER Hsp70 BiP (also known as HSPA5) (Hosoda et al., 2003). ERdj5 functions to reduce non-native disulfides prior to their targeting for degradation (Ushioda et al., 2008), and allows correct folding of specific disulfide-bonded proteins (Moustardas et al., 2021; Oka et al., 2013). ERdj5 has multiple active sites, some of which have relatively low reduction potential (Hagiwara et al., 2011) suggesting a role during reduction rather than formation of disulfides.

The relative roles of ERp57 and ERdj5 as oxidases, reductases or isomerases, and their substrate specificity are still poorly characterised. We suggest that they might act as conduits for the transfer of reducing equivalents from the ER membrane either directly to reduce specific substrates or indirectly via the network of PDI enzymes (Fig. 1C). In this study, we assess whether ERp57 and ERdj5 require the cytosolic thioredoxin reductive pathway for activity, and whether they are individually or together required for correct disulfide bonding in specific substrates.

We have previously developed an *in vitro* translation system where we can induce the ER-reductive pathway and assess its impact on folding of newly synthesised proteins (Cao et al., 2020; Poet et al., 2017; Robinson et al., 2020). Here we supplement this translation system with semi-permeabilised cells (SP cells) made from ERp57- and ERdj5-knockout (KO) cell lines to determine the role of these two proteins in the folding of different proteins. Our results demonstrate that ERp57 is directly involved in the isomerisation of non-native disulfides during the folding of specific proteins. In the absence of ERp57, ERdj5 acts as a reductase either directly or via alternative isomerases. Neither enzyme is essential for disulfide formation or for the transfer of reducing equivalents into the oxidoreductase network. These results highlight the diverse, yet substrate-specific, pathways that exist to provide a robust system for the reduction of non-native disulfide bonds in folding proteins.

## RESULTS

### Experimental design

A rabbit reticulocyte lysate (RRL) translation system that can be programmed with RNA templates to produce radiolabelled translation product was used to study disulfide formation and folding of newly synthesised proteins (Fig. 1D). This system was supplemented with either microsomes or SP cells as a source of ER, which allowed for disulfide formation in translocated proteins. If RNA templates that did not have a stop codon were added to the reaction, then stalled translation intermediates were produced, allowing folding events that take place before translocation was complete to be assessed. If stop codons were present in the template, then the substrate was released from the ribosome on completion of translation.

Commercial RRL that is air equilibrated (oxidising lysate; Ox) and without supplements is sufficiently oxidising so that disulfide bonds formed in both untranslocated and translocated proteins (Fig. 1E). If glucose 6-phosphate (G6P) was added to the lysate (balanced lysate; Bal) this enhanced the pathway described in Fig. 1A, and the lysate becames sufficiently reducing so that disulfides did not form in untranslocated proteins, whereas disulfide formation, reduction and isomerisation does occur in proteins translocated into the ER. If DTT is added to the system (reducing lysate; Red) then both the lysate and the ER became reducing, preventing disulfide formation in both untranslocated and translocated proteins. By comparing results in these three lysates the involvement of the ER reductive pathway in the folding of different substrates can be determined.

### β1-integrin folds into a native structure under conditions that favour disulfide isomerisation

**Fig. 2.**
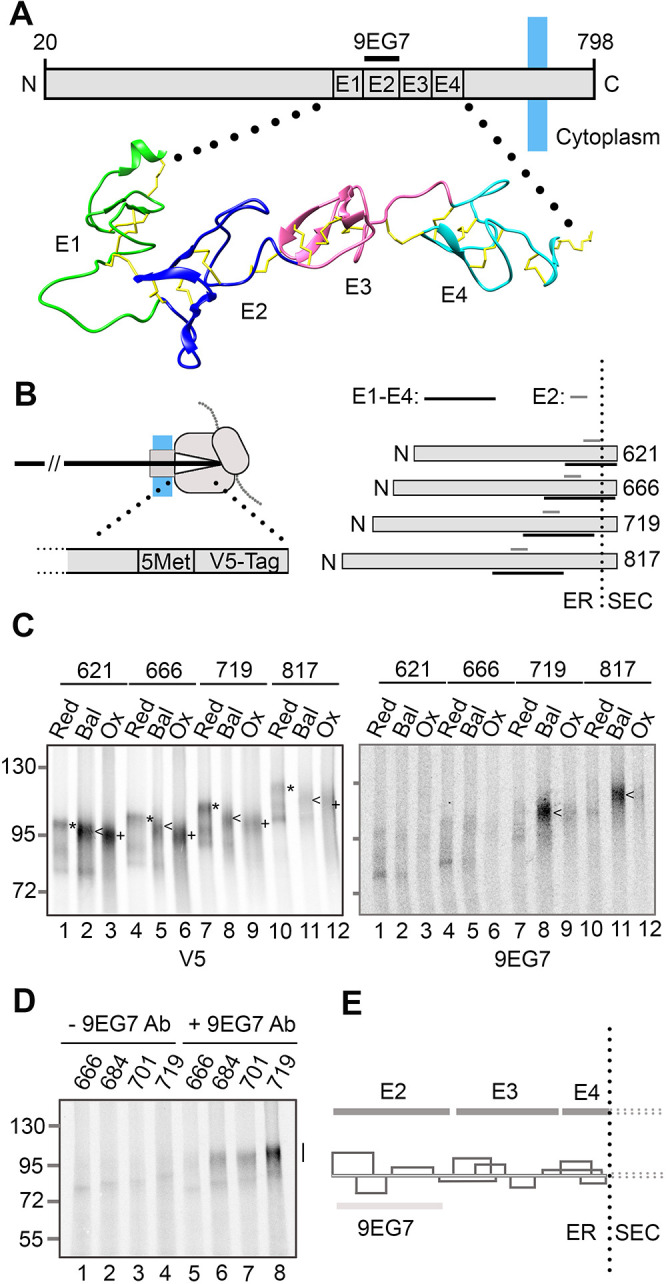
**The 9EG7 epitope of β1-integrin forms before translocation is complete and requires ER exposure of downstream sequence.** (A) Topology diagram of β1-integrin highlighting the location of the EGF domains (E1–E4) and the 9EG7 conformational epitope. The ribbon diagram (PDB: 7NXD) shows the structure and disulfide bonding of the EGF domains. (B) Diagram (left) of a stalled β1-integrin translation intermediate with a 5Met–V5 tag extension at the C-terminus. The diagram (right) shows the estimated N-terminal ER exposure of selected translation intermediates (621–817) with the position of the EGF domains (E1–E4) highlighted. SEC, the Sec61 translocon. (C) Non-reducing SDS-PAGE of radiolabelled translation intermediates (621–817 amino acids long) translated in either reducing (Red), redox balanced (Bal), or oxidising (Ox) lysates and immunoisolated with either V5 (left panel) or 9EG7 (right panel) antibodies. Annotations highlight immunoisolated translation product from reducing (*) redox balanced (<) and oxidising (+) lysates. (D) Non-reducing SDS-PAGE of translation intermediates (666–719 aa) translated in redox-balanced lysates and immunoisolated using the 9EG7 antibody (lanes 5–8) in comparison to protein G–Sepharose beads alone (lanes 1–4). The vertical bar highlights the gel position of the 9EG7 immunoisolated material in lanes 6–8. (E) A cartoon of a β1-integrin translation intermediate, at the minimal length required for formation of the 9EG7 conformational epitope. The diagram (bottom) shows the native disulfide connectivity in this exposed region. The location of the E2–E4 domains and the 9EG7 epitope is as indicated. All experiments shown in this figure have been repeated twice (*n*=3) from independent translation reactions and representative data are shown.

β1-integrin is a large transmembrane glycoprotein with a cysteine-rich EGF repeat region (E1–E4, Fig. 2A). It contains a conformation-specific epitope (9EG7) located in the E2 domain (Askari et al., 2010; Su et al., 2016), which is present in the native protein (Bazzoni et al., 1995) but is lost on disulfide reduction (Tiwari et al., 2011). To investigate whether folding to the native form can occur during translocation of β1-integrin, we expressed stalled translation intermediates of increasing lengths in the different lysates containing microsomes, and monitored the presence of the 9EG7 epitope. These intermediates were generated with a C-terminal V5 tag to enable isolation of the total translation product for comparison (Fig. 2B, left panel). Translation intermediates were selected between 621 and 817 amino acids in length (numbering from the start codon) to enable incremental exposure of the EGF repeat region (Fig. 2B, right panel). On non-reducing SDS-PAGE, each V5 isolated intermediate translated in reducing lysate ran as a slow migrating band (marked by *) corresponding to reduced protein (Fig. 2C, left panel). The intermediates produced in balanced lysate ran faster (marked by <) than the reduced protein, which indicates that extensive disulfide formation occurred with all constructs. The intermediates produced in the oxidising lysate (marked by +) ran even faster than the equivalent samples produced in the balanced lysate, which indicates that different long-range disulfide configurations were formed. This illustrates how non-native disulfides can form between cysteine residues that are widely separated in the amino acid sequence and are not necessarily limited to cysteine residues that are closer in the sequence, an occurrence that has also been observed for LDLr (Jansens et al., 2002). When the same samples were immunoisolated using the 9EG7 antibody (Fig. 2C, right panel), translation product was only detected for the 719 and 817 intermediates in the balanced lysate (lanes 8 and 11; <). This demonstrates that, at the longer intermediate lengths, β1-integrin can fold to form the 9EG7 epitope, but only if the ER reductive pathway is active to promote disulfide isomerisation.

To define more precisely the point in translocation at which the 9EG7 epitope forms, intermediates between 666 and 719 amino acids in length were generated in the balanced lysate (Fig. 2D). These samples were immunoisolated with the 9EG7 antibody (lanes 5–8) and compared to the same samples without the antibody as a background control (lanes 1–4). The 684 intermediate (lane 6) is the first point at which the 9EG7 epitope is recognised by the antibody. If ∼63 amino acids are sequestered in the ribosome–Sec61 complex (Robinson et al., 2017), all the E1, E2 and E3 and part of the E4 domain is ER exposed in the 684 intermediate. This reasoning indicates that at this chain length, the cysteine residues in the E3 and E4 domain required for maturation of the 9EG7 epitope are translocated into the ER lumen (Fig. 2E).

### The folding of β1-integrin is assisted by ERp57 and ERdj5 cannot compensate for loss of this activity

**Fig. 3.**
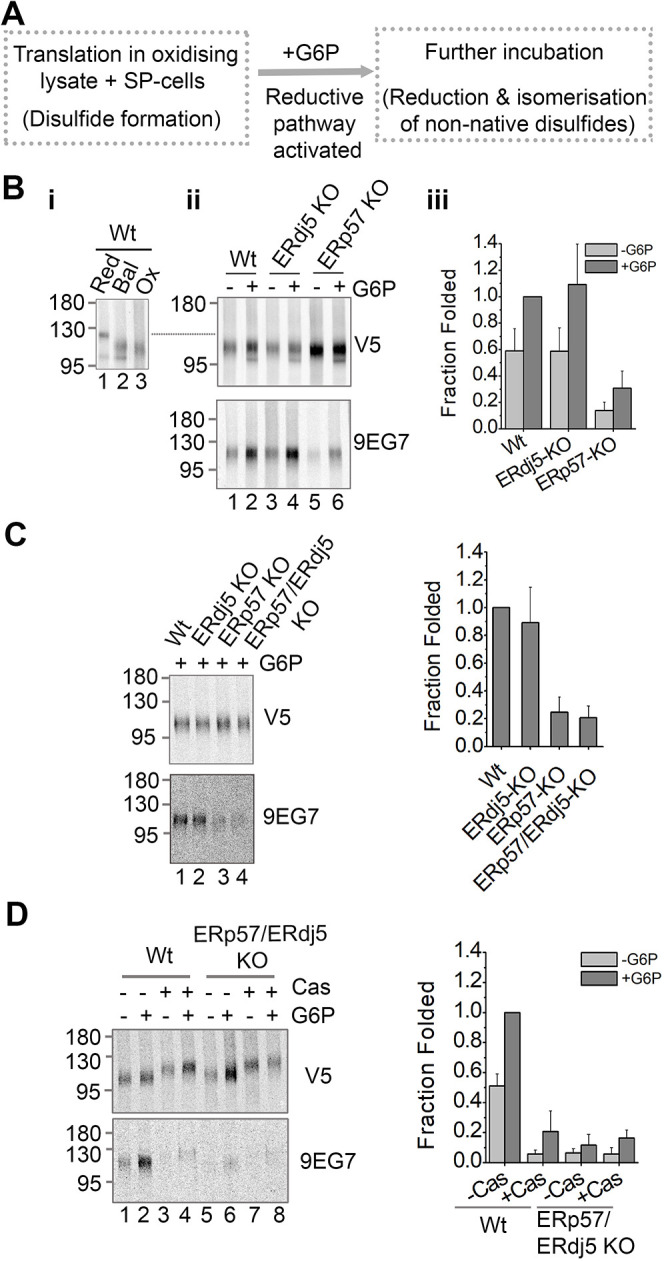
**ERp57 knockout prevents the efficient folding of β1-integrin, whereas ERdj5 knockout has no impact on folding.** (A) Experimental setup to assay disulfide isomerisation in translocated proteins. (B–D) Non-reducing SDS-PAGE of radiolabelled β1-integrin intermediate 817. (Bi) Translations were performed with reducing (Red), balanced (Bal), or oxidising (Ox) lysates. (Bii,C,D) Translations were performed with the indicated cell lines in oxidising lysate and then recovered with or without G6P. Castanospermine (Cas) was added where indicated. Samples were immunoisolated with a V5 or 9EG7 antibody. The bar charts to the right of each gel set show the fraction of β1-integrin folded as estimated by quantifying gel bands. For this purpose, each value was normalised to the wild-type (Wt) +G6P sample. The 9EG7 samples (folded protein) were then divided by the equivalent V5 samples (total protein) to calculate fraction folded [mean±s.d. for *n*=5 (B), *n*=3 (C) and *n*=4 (D)].

Having demonstrated that formation of the 9EG7 epitope requires a defined length of sequence translocation into the ER and the presence of an active reductive pathway, we were well placed to determine the oxidoreductase requirements for correct disulfide formation. It has been shown that ERp57 assists in correct disulfide formation during folding of β1-integrin, possibly acting as an isomerase (Jessop et al., 2007, 2009). ERdj5 has also been identified as a mixed disulfide partner of β1-integrin in proteomic screens (Oka et al., 2013), so might well act as an isomerase during folding or as a reductase during degradation of β1-integrin. To evaluate the role of each enzyme during the resolution of non-native disulfides within β1-integrin, we performed translations supplemented with SP cells prepared from either ERdj5- or ERp57-KO cells (van Lith et al., 2021). The β1-integrin 817 intermediate was first synthesised in an oxidising lysate, to promote the formation of non-native disulfides, and then disulfide isomerisation was induced, by the addition of G6P (Fig. 3A). Control reactions without G6P addition were performed for comparison.

Neither the ERdj5 KO nor the ERp57 KO prevented disulfide formation in the β1-integrin intermediate as judged by the increased mobility of the non-reduced compared to the reduced V5 samples (Fig. 3Bii, compare mobilities to lane 1, Fig. 3Bi, dotted line added for guidance). Immunoisolation with the 9EG7 antibody revealed a degree of native folding under −G6P conditions (Fig. 3Bii, lanes 1, 3 and 5, 9EG7), which contrasts to the results in Fig. 2. This discrepancy is likely to be a result of the longer translation time (60 mins compared to 30 mins) and the use of SP cells, which are known to support more efficient folding than microsomes (Wilson et al., 1995). Nevertheless, the addition of G6P increased the amount of folded protein found in each reaction (Fig. 3Bii, compare lanes 2, 4 and 6, and lanes 1, 3 and 5, 9EG7). Quantification revealed that the amount of folded protein in each sample approximately doubled when G6P was added (Fig. 3Biii). Together, these results demonstrate that although a fraction of the β1-integrin synthesised can fold in the oxidising lysate, presumably due to residual G6P or NADPH, disulfide reduction and isomerisation induced by added G6P dramatically increases the yield of folded protein. There was no difference between the yield of correctly folded protein with ERdj5-KO SP cells compared to wild-type cells (Fig. 3Biii). In contrast, there was a large decrease in the fraction of folded protein with ERp57-KO SP cells compared to the wild-type samples both in the presence and absence of G6P. These results demonstrate that ERp57 is essential for efficient isomerisation of non-native disulfides within β1-integrin and show that ERdj5 has no essential role in this process.

As both ERp57 and ERdj5 have reductase activity *in vitro* (Frickel et al., 2004; Ushioda et al., 2008), it is possible that loss of ERdj5 activity is compensated for by ERp57 thereby masking any effect on isomerase activity towards β1-integrin. To see whether the absence of both enzymes further influenced the disulfide rearrangement within β1-integrin, we performed translations with ERdj5/ERp57 double KO SP cells (van Lith et al., 2021). The translation product from the ERp57/ERdj5-KO sample, showed a similar level of folding to the ERp57-KO (Fig. 3C, lanes 3 and 4). Removing both enzymes did not cause any additional changes in the isomerisation of non-native disulfides in β1-integrin compared with those seen upon removing ERp57 alone. Taken together these results indicate that ERdj5 does not influence the folding of β1-integrin even in the absence of ERp57.

Previous work has shown that entry into the calnexin cycle is required for ERp57 to assist in the folding of β1-integrin (Jessop et al., 2009). To confirm that this is the case in this system we assessed folding of β1-integrin-817 in the presence of castanospermine. This is a glucosidase inhibitor that prevents entry of substrates into the calnexin cycle by inhibiting trimming of oligosaccharide side chains. The addition of castanospermine did not influence the expression of β1-integrin-817 (Fig. 3D, V5), but a slower gel migration is observed due to the additional glucose residues (Fig. 3D, V5 lanes 3,4 and 7,8). In translations containing wild-type SP cells, the formation of the 9EG7 epitope was inhibited when castanospermine was present (Fig. 3D, lanes 1–4). Quantification revealed the levels of folding in castanospermine samples to be equivalent to those observed in the ERp57/ERdj5 double KO. This confirms that entry into the calnexin cycle is required for ERp57 to enhance the correct folding of β1-integrin.

### Disulfide isomerisation in the disintegrin domain takes place in the absence of ERdj5 or ERp57

**Fig. 4.**
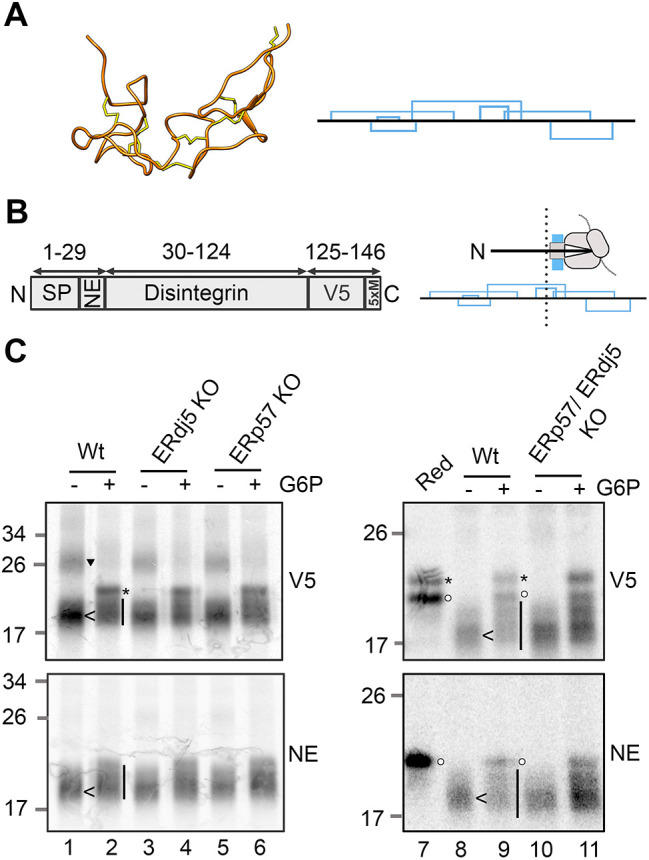
**Disulfide rearrangements in the disintegrin domain of ADAM10 are independent of ERdj5 and ERp57.** (A) Ribbon diagram of the ADAM10 disintegrin domain (left) with disulfide bonds in yellow, drawn using PDB file 6BE6. The diagram (right) shows the disulfide connectivity. (B) Topology diagram of the disintegrin 146 construct (left) showing the signal peptide (SP), neo-epitope tag (NE), the disintegrin domain sequence (Disintegrin), the V5 sequence and five methionine residues. The ribosome diagram (right) shows the expected ER exposure of the stalled disintegrin 146 intermediate in terms of the cysteine residues that make up the disulfide bonds. (C) Non-reducing SDS-PAGE of the radiolabelled disintegrin 146 intermediate. Translations were performed with the indicated cell lines in oxidising lysate and then recovered with or without G6P. Samples were immunoisolated using either a V5 or neoepitope (NE) antibody. A reduced control is also shown (Red). Wt, wild-type. Representative data is shown in this figure from at least three independent repeats. Annotations highlight immunoisolated reduced preprotein (*) or mature protein (○), and disulfide-bonded monomers (<) or dimers (▼). The vertical line highlights a smear of disulfide-bonded protein.

We next studied disulfide rearrangements in the disintegrin domain of ADAM10. This short domain is not glycosylated and has a high density of cysteine residues and complex disulfide bonding (Fig. 4A). Previous studies with partially translocated intermediates of this protein, found that disulfide isomerisation is dependent on activation of the cytosolic reducing pathway (Robinson et al., 2020). The disintegrin domain construct has an N-terminal signal peptide (SP) with a neoepitope tag (NE) and a C-terminal V5 tag (Fig. 4B). The neoepitope is only recognised following signal peptide cleavage. The stalled 146 intermediate used in this study is partially ER exposed (Fig. 4B, right), which promotes the formation of non-native disulfide bonds. When separated on non-reducing SDS-PAGE the 146 intermediate has different banding patterns when expressed in the oxidised, reducing or balanced lysates (Robinson et al., 2020). In the current study, the same intermediate was first translated in oxidising lysate with SP cells and then G6P was added to activate the cytosolic reducing pathway and induce disulfide isomerisation.

When the disintegrin domain was expressed in reducing lysate (Fig. 4C, lane 7) and immunoisolated with the V5 antibody, two bands were present corresponding to reduced preprotein (marked by *) and reduced mature protein (marked by a circle). The same sample isolated with the neo-epitope antibody showed a single band corresponding to reduced mature protein (circle). The samples expressed in oxidising lysate with wild-type SP cells (lanes 1 and 8) show a faster migrating band (marked by <), which corresponds to disulfide-bonded translocated protein, and also a slower migrating band (triangle) indicating disulfide-bonded dimers. On addition of G6P (lanes 2 and 9) a prominent band appears (*), which is specific to the V5 samples, corresponding to reduced untranslocated protein. The dimer band also disappears and there is a smear (vertical line) below the preprotein band, which is present in the V5 and NE isolated samples. This distinctive change in the gel banding pattern on the addition of G6P reflects the isomerisation of non-native disulfides. Translations containing SP cells made from the ERdj5 (lanes 3 and 4) and ERp57 (lanes 5 and 6) KO cell lines, showed the same gel band patterns as the samples with wild-type SP cells indicating that disulfides still form and are isomerised in the absence of these enzymes. The same band patterns were also observed for translations containing SP cells made from the double KO (lanes 10 and 11). Together, these results indicate that disulfide formation and isomerisation take place in the stalled disintegrin nascent chain independently of both ERdj5 and ERp57 suggesting an alternative pathway to ensure the correct disulfide rearrangements in this protein. Note that this alternative pathway is dependent on the cytosolic reductive pathway as it requires the addition of G6P for isomerisation.

### Both ERp57 and ERdj5 influence the folding of LDLr

**Fig. 5.**
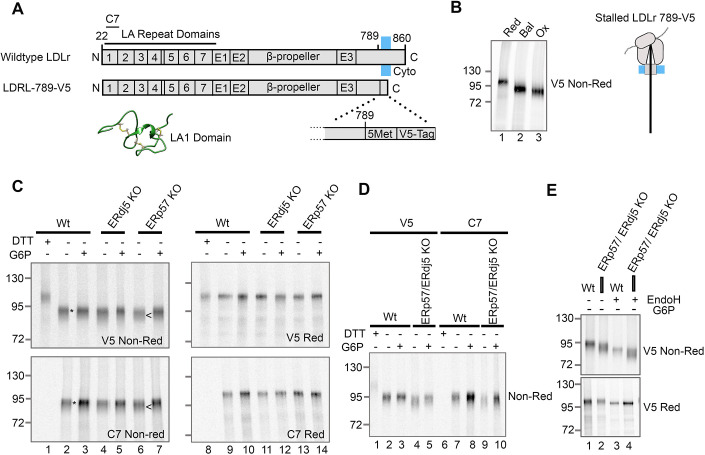
**Knockout of ERdj5 and ERp57 together disrupts the folding of LDLr.** (A) Topology diagram of wild-type LDLr, showing the location of the LA repeat domains (1–7), the EGF domains (E1, E2 and E3) and the β-propeller domain. The location of the C7 epitope is also indicated. The topology diagram of the truncated construct used in this study (LDLr-789-V5), consisting of LDLr residues 1–789 with the addition of five methionine residues and a V5 tag at the C-terminus. The green ribbon diagram shows the structure of the LA1 domain with disulfide bonds in yellow, drawn using PDB file 1LDL. (B) Non-reducing SDS-PAGE of radiolabelled LDLr-789-V5 produced as a stalled intermediate (diagram right) in translation reactions containing microsomes and either reduced (Red), balanced (Bal) or oxidised (Ox) lysates. (C–E) Non-reducing (non-Red) or reducing (Red) SDS-PAGE showing radiolabelled translation product of stalled LDLr-789-V5 translated with the indicated cell lines in oxidising lysate and recovered with or without G6P as indicated. Control samples translated in reducing lysate (+DTT) are also shown. Samples were immunoisolated using a V5 or C7 antibody. Annotations highlight differences between the mobility of translation products formed in the absence of G6P and the presence of Wt (*) or ERp57 KO (<) cells. In E, samples are EndoH treated as indicated. Wt, wild-type. All gels in this figure are representative of at least three independent experiments.

The low-density lipoprotein receptor (LDLr) is a large transmembrane glycoprotein, which undergoes extensive disulfide rearrangements during folding. It contains disulfide-rich tandem repeats at the N-terminus and three EGF domains (Fig. 5A). A conformational-specific antibody (C7) recognises an epitope in the LA1 repeat that is dependent on disulfide formation. The nuclear magnetic resonance (NMR) structure of the LA1 repeat highlights the dense disulfide bonding in the repeat domains (Daly et al., 1995). In this series of experiments, we used a truncated construct, consisting of the entire ectodomain of LDLr with a C-terminal V5 tag (LDLr-789-V5) (Fig. 5A). This construct was initially expressed as a stalled translation intermediate (Fig. 5B) in the translation system containing microsomes. When synthesised in reducing lysate (lane 1), the translation product separated as a single product. In the balanced lysate, the translation product migrated faster than the reduced protein (lane 2) indicating disulfide formation, and when synthesised in the oxidising lysate it migrated slightly faster still, indicative of non-native disulfides (lane 3), as described previously (Jansens et al., 2002). This represents the various disulfide configurations of LDLr that form in the different lysates.

Next, we investigated the role of ERp57 and ERdj5 in the folding of LDLr, by synthesising the LDLr-789-V5 nascent chain in the presence of SP cells made from the KO cell lines. LDLr-789-V5 was synthesised in an oxidising lysate, then G6P was added to initiate disulfide isomerisation. Consistent with the results from the experiment with microsomes, the samples with G6P ran slightly slower on SDS-PAGE and formed a tighter gel band than the equivalent samples without G6P (Fig. 5C). There was no difference between the mobility of LDLr synthesised with wild-type or ERdj5 KO cells but there was a slight difference in the electrophoretic mobility with the ERp57 KO cells translated without G6P [compare lanes 2 (*) and 6 (<)]. This indicates that ERp57 is required for disulfide rearrangements during LDLr folding. The C7 antibody recognised LDLr in all samples (C7, non-Red, lanes 2–7) except for the reduced control (lane 1), consistent with previous studies indicating that the folding of the LA1 domain occurs early in LDLr translocation (Kadokura et al., 2020). Samples run under reducing conditions showed no difference in mobility between lanes, and so the differences in gel mobility observed in the non-reducing gels are due to disulfide formation. The mobility differences of the samples separated under reducing and non-reducing conditions demonstrate disulfide formation (correct or incorrect) in the absence of either ERdj5 or ERp57.

The same G6P recovery experiment, using LDLr-789-V5, was performed in reactions containing ERp57/ERdj5 double KO SP cells (Fig. 5D). These samples had a noticeably faster electrophoretic mobility than the equivalent wild-type samples, both in the absence (compare lanes 2 and 4) and presence of G6P (compare lanes 3 and 5). The same changes in mobility were also seen in the samples isolated with the C7 antibody (compare lanes 7 and 9 and lanes 8 and 10). For a clearer comparison of the mobility changes that result from the double knockout of ERp57 and ERdj5, LDLr-789-V5 was translated in an oxidising lysate and samples run in adjacent gel lanes (Fig. 5E, upper panel, lanes 1,2). The dramatic change in mobility is very clear under these conditions. To confirm that this change was not influenced by glycosylation, we also treated samples with EndoH before electrophoresis. EndoH removes glycans, and so the treated samples run faster through the gel (lanes 3,4) than untreated samples (lanes 1,2). The difference in mobility between wild-type and ERp57/ERdj5 double KO samples, is maintained despite the removal of glycans (lanes 3,4, upper panel). Furthermore, when run under reducing conditions (Fig. 5E, V5 red) there was no change in electrophoretic mobility between the wild-type and ERp57/ERdj5 double KO samples. Together, these results confirm that the electrophoretic mobility change observed is a result of the altered disulfide configuration of LDLr-789-V5 when both ERp57 and ERdj5 are knocked out. This change appears more prominent than that observed for the single ERp57 knockout in Fig. 5C, suggesting that both ERp57 and ERdj5 have a role in the folding of LDLr.

### ERp57 promotes correct folding of LDLr in the absence of added G6P

**Fig. 6.**
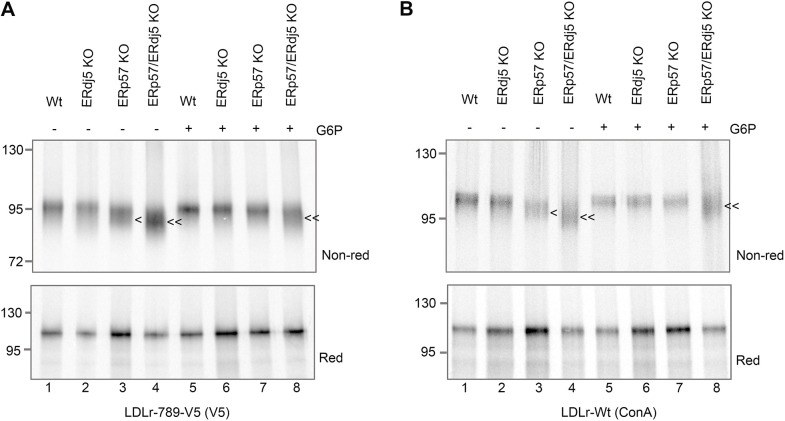
**The cytosolic reducing pathway supplies reducing power to ERp57 and ERdj5 to enable efficient folding of LDLr, but both enzymes also influence folding when the pathway is inactive.** Phosphoimages of SDS-PAGE gels run under reducing (Red) or non reducing (Non-red) conditions showing radiolabelled translation product of (A) LDLr-789-V5 and (B) LDLr-Wt translated with SP cells made from the indicated cell lines. Samples were initially translated in oxidising lysate before addition of G6P after 30 mins to +G6P samples as indicated. Samples were either immunoisolated using a V5 antibody or isolated using ConA beads. Annotations highlight the increased mobility of translation products formed in the presence of ERp57 KO (<) or ERp57/ERdj5 double KO (<<) cells. Wt, wild-type. All gels are representative of at least three independent experiments.

In the previous section, the results demonstrate that the joint loss of ERp57 and ERdj5 creates a different disulfide configuration for LDLr-789-V5 to that seen upon the loss of either enzyme alone. To provide a direct comparison of how LDLr-789-V5 folding differs between the cell types, we performed translations simultaneously using the G6P recovery method. Samples were isolated by V5-immunoisolation and ran adjacently on SDS-PAGE gels, to detect subtle changes in gel mobility (Fig. 6). When G6P was not included during translation, LDLr synthesised with ERdj5-KO SP cells (Fig. 6A, lane 2) had the same mobility as that synthesised with wild-type SP cells (Fig. 6A, lane 1). In contrast, the electrophoretic mobility of the LDLr synthesised in the presence of ERp57 KO SP cells was clearly increased (marked by <; lane 3). These results confirm that in the absence of added G6P, ERp57 has an indispensable role in the folding of LDLr. The LDLr synthesised with ERp57/ERdj5 KO SP cells migrates even faster (marked by <<; lane 4) than the ERp57-KO sample (marked by <; lane 3), suggesting that ERdj5 also modulates the folding of LDLr in the absence of added G6P, but this activity is not sufficient to restore a wild-type phenotype in ERp57-KO samples. The fact that a change in gel mobility is not observed for the single ERdj5 KO (lane 2), means that ERp57 alone is sufficient to recover any loss of ERdj5 activity. The requirement for ERdj5 seems to be limited to when ERp57 is absent.

### In the presence of added G6P, ERdj5 can compensate for the loss of ERp57 to ensure LDLr folds correctly

On adding G6P to the wild-type, ERdj5-KO and ERp57-KO samples, LDLr-789 V5 migrates as a single band (Fig. 6A, lanes 5–7), whereas the ERp57/ERdj5 double KO sample retains its distinctive fast migration (lane 8; marked by <<). On the reducing gels (Fig. 6A, Red) there were no changes in electrophoretic mobility when comparing the different samples, and so any changes detected on the non-reducing gels can be attributed solely to changes in disulfide bonding. The restoration of the wild-type phenotype for the ERp57-KO shows that an alternative pathway can compensate for the loss of ERp57 activity in the presence of an optimal cytosolic reducing pathway. The folding of LDLr when both ERp57 and ERdj5 are absent, remains disrupted despite an active reducing pathway, indicating that ERdj5 is required to maintain a functional reducing pathway for LDLr isomerisation.

To determine whether the same results are observed with wild-type LDLr we translated the full-length sequence, which contains a stop codon so that the protein is released and integrated into the ER membrane. The experiments were performed under the same conditions but this time the translation product was isolated using concanavalin A beads, which bind glycosylated and therefore translocated protein. The same changes in gel mobility were observed with these full-length LDLr samples (Fig. 6B) as with the stalled construct. This confirms that the changes in disulfide formation caused by the knockout of ERp57 and ERdj5 are relevant to the folding of the full-length protein.

## DISCUSSION

**Fig. 7.**
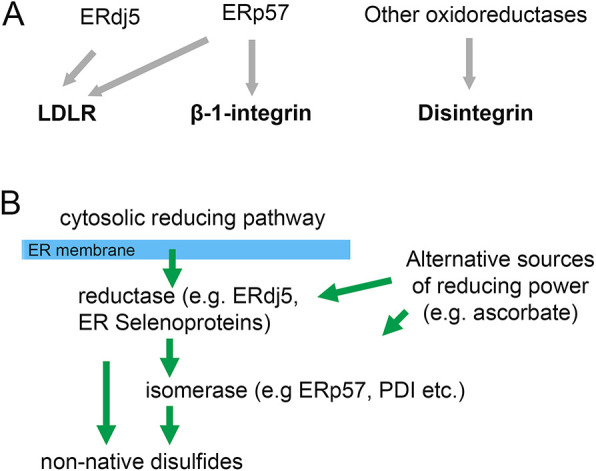
**A model for the role of ERp57 and ERdj5 in the ER reducing pathway.** (A) The correct folding of different substrates requires specific oxidoreductases. LDLr and β1-integrin are glycoproteins, whereas the disintegrin domain is not glycosylated. (B) The cytosolic reducing pathway is linked to isomerases such as ERp57 in the ER via reductases such as ERdj5 or ER selenoproteins. These reductases might well reduce folding proteins as well as other PDI family members. Other electron doners, such as ascorbate, might contribute to the reduction of ER proteins.

The ability to manipulate the ER reductive pathway using a cell-free translation system has enabled us to dissect the process of disulfide bond formation, reduction and isomerisation within three representative heavily disulfide-bonded protein substrates. We focused on the isomerisation of non-native disulfides, addressing the requirement for a robust reductive pathway to drive initial disulfide reduction by PDI family members. The findings are summarised in a schematic (Fig. 7A) illustrating that ERp57 is required for the efficient isomerisation of non-native disulfides in glycoproteins, whereas alternative oxidoreductases can isomerise disulfides in non-glycoproteins. We show that ERdj5 alone is not required for the isomerisation of non-native disulfides but is required in the absence of ERp57, ensuring the efficient function of alternative isomerases during LDLr maturation. In this regard, it acts as a reductase, either linking the cytosolic reductive pathway with the PDI family network, or by acting directly on LDLr to reduce non-native disulfides, a role that is consistent with its low redox potential (Hagiwara et al., 2011; Ushioda et al., 2008) (Fig. 7B). Neither ERp57 nor ERdj5 are required for disulfide formation, a reaction that is most likely carried out by other PDIs.

The essential requirement for ERp57 in correct folding of β1-integrin, even in the presence of a robust reductive pathway, demonstrates that other enzymes cannot compensate for ERp57 function in the folding of this glycoprotein. In contrast, the observed requirement for ERp57 in the folding of LDLr only occurs in the absence of added G6P illustrating that, in the presence of a robust reductive pathway, the role of ERp57 is less important. The alternative isomerase activity that functions during LDLr folding in the presence of G6P was compromised in the absence of ERdj5 indicating the essential role of this enzyme. Within cells it has been shown that depleting ERdj5 compromises correct disulfide formation and secretion of LDLr (Oka et al., 2013) and members of the Kallikrein family (Moustardas et al., 2021). ERdj5 was shown to form mixed disulfides directly with the LDLr, suggesting that it can directly reduce disulfides within this substrate to facilitate correct folding. Our results do not preclude that possibility, but do suggest that ERp57 can fulfil the essential isomerase role within our experimental system. The reason why ERdj5 assists in the folding of LDLr but not the other substrates studied here remains unknown. Binding studies have shown that ERdj5 recognises aggregation-prone sequences and is more selective towards its substrates than BiP (Behnke et al., 2016). However, an interaction between β1-integrin and ERdj5 has been detected in cells (Oka et al., 2013), which indicates ERdj5 has affinity to both proteins despite only influencing the folding of one. Future studies are therefore required to elucidate the mechanisms by which ERdj5 selectively reduces folding proteins. The results with β1-integrin, LDLr and the disintegrin domain suggests the existence of a complex system of disulfide exchange enzymes within the ER that can adapt to allow correct disulfide formation in a range of protein substrates.

The alternative isomerase capable of catalysing disulfide exchange in non-glycoproteins, such as the disintegrin domain, is still dependent on a robust reductive pathway. In this case, the isomerase is independent of ERdj5. This suggests that there is not an obligatory requirement for ERdj5 to act as an intermediary in the transfer of reducing equivalents from the ER membrane (Fig. 7B). Other candidates for this purpose include the ER-resident selenoproteins (Pitts and Hoffmann, 2018; Zhang et al., 2020), some of which are oxidoreductases with redox properties that favour disulfide reduction (Hondal et al., 2013). To date the exact function of the ER selenoproteins has been difficult to ascertain (Addinsall et al., 2018), however, Selenoprotein F (Sep15, also known as SELENOF) has been shown to be involved in secretion of functional immunoglobulins, consistent with a role in redox quality control (Yim et al., 2018) and Selenoprotein N can act as a reductase regulating ER Ca^2+^ homeostasis (Chernorudskiy et al., 2020). In addition, canonical PDI (PDIA1, also known as P4HB) is known to act as an isomerase *in vitro* (Hawkins et al., 1991) and in yeast (Laboissiere et al., 1995), and has a redox potential suited to both reduction and oxidation (Chambers et al., 2010) making it a likely candidate for the isomerase required for non-glycoproteins.

The requirement for isomerase activity is particularly acute for the substrates we have chosen and the approach taken, due to promiscuous disulfide formation within the specific untranslocated nascent chains. The β1-integrin intermediates formed disulfides but required exposure of the EGF domains for the individual domains to fold correctly and attain their native interdomain disulfides. LDLr required exposure of most of the ectodomain to allow the formation of its native disulfides, as shown previously in a study that demonstrated very early disulfide formation in the nascent chains synthesised in cultured cells (Kadokura et al., 2020). Finally, the disintegrin domain also formed disulfides during translocation but requires isomerase activity to complete folding (Robinson et al., 2020). The use of stalled translocation intermediates enabled us to demonstrate that these rearrangements can take place while the nascent chain is still associated with the ER translocon. It is well established that oxidoreductases can form mixed disulfides with translocating proteins (Molinari and Helenius, 1999) and it was recently shown that the PDI family member ERp46 (also known as TXNDC5) can introduce disulfides into nascent chains efficiently at an early stage of translocation (Hirayama et al., 2021). Our results are consistent with oxidoreductases introducing disulfides as a nascent protein emerges into the ER with subsequent isomerisation of non-native disulfides.

Although our understanding of how the ER-reducing pathway functions and its role in protein folding has greatly improved, there are many pathway components that remain unidentified. These include the membrane protein that transfers reducing equivalents from the cytosol to the ER and the mechanism of transfer of these equivalents to the ER oxidoreductases. Although our focus has been on transfer of reducing equivalents by disulfide exchange, there remains the possibility that alternative electron donors could provide reducing equivalents to the PDIs (Fig. 7B). One potential source would be ascorbate, which is known to become depleted in mice with hyper-sulfenylation of protein thiols (Zito et al., 2012). Just like the multiple pathways for thiol oxidation, it appears that reduction of non-native disulfides will require multiple pathways feeding through the PDI family of disulfide exchange proteins.

## MATERIALS AND METHODS

### Software

Molecular graphics images were drawn with UCSF Chimera version 1.13.1 (Pettersen et al., 2004). Quantification of gel band intensities was performed using ImageJ 1.52S (Schneider et al., 2012). Histograms were prepared using Origin 6.0 professional software.

### Template generation

Templates encoding β1-integrin intermediates were PCR amplified from the human β1-integrin sequence in the pSPUTK vector (Tiwari et al., 2011) using the same forward primer (T7 β-1-Integrin, 5′-GATGGCTAATACGACTCACTATAGGGTCAGGCCACCATGAATTTACAACCAATTTTCT-3′) and different reverse primers corresponding to the amino acid length of each intermediate (621, 5′-GGTGCTGTCCAGGCCCAGCAGAGGGTTGGGGATGGGCTTGCCCATCATCATCATCATGCTGGCTTCACAAGTACTAGTATCCAAAG-3′; 666, 5′-GGTGCTGTCCAGGCCCAGCAGAGGGTTGGGGATGGGCTTGCCCATCATCATCATCATAACACATTCTTTATGCTCAGCACAGACACC-3′; 684, 5′-GGTGCTGTCCAGGCCCAGCAGAGGGTTGGGGATGGGCTTGCCCATCATCATCATCATACATTCCTGTGTGCATGTGTCTTTCTTTTCTC-3′; 701, 5′-GGTGCTGTCCAGGCCCAGCAGAGGGTTGGGGATGGGCTTGCCCATCATCATCATCATCGGCTGGGGTAATTTGTCCCGACTTTCTAC; 719, 5′-GGTGCTGTCCAGGCCCAGCAGAGGGTTGGGGATGGGCTTGCCCATCATCATCATCATCCAACAGTCGTCAACATCCTTCTCCTTACAATGG-3′; and 817, 5′-GGTGCTGTCCAGGCCCAGCAGAGGGTTGGGGATGGGCTTGCCCATCATCATCATCATTTTTCCCTCATACTTCGGATTGACCACAGTTGTTAC-3′).

The templates encoding the disintegrin 146 intermediate were PCR amplified from the extended-disintegrin AST construct (Robinson et al., 2020), (forward primer, 5′-GATGGCTAATACGACTCACTATAGGGTCAGGCCACCATGAGCAGATCTGTGGCCCTGG-3′; reverse primer: 5′-CATCATCATCATCATGGTGCTGTC-3′).

The template encoding the LDLr 789-V5 intermediate was PCR amplified from the human LDLr cDNA sequence in pcDNA 3.1 (Oka et al., 2013) (forward primer, T7: 5′-TAATACGACTCACTATAGGG-3′; Reverse primer, LDLR 789-V5: 5′-GGTGCTGTCCAGGCCCAGCAGAGGGTTGGGGATGGGCTTGCCCATCATCATCATCATAGCCCTCACGCTACTGGGCTTCTTC-3′).

For template encoding full-length LDLr, the LDLr-pcDNA 3.1 plasmid was linearised by NotI (NEB) cleavage. The resulting PCR/plasmid templates were ethanol precipitated, resuspended in nuclease free water, and transcribed into RNA using T7 RNA polymerase (Promega), 37°C, 2 h. The RNA was then ethanol precipitated, resuspended in nuclease free water and stored at −80°C.

### Preparation of microsomes and SP cells

Dog pancreas microsomes (referred to as microsomes) were a gift from Prof. Arthur E. Johnson, Texas A&M University, USA, and were originally made according to the methods of Walter and Blobel (Walter and Blobel, 1983). Microsomes were treated with micrococcal nuclease from *Staphylococcus aureus* (Sigma) before use. For this purpose, nuclease (10 μg/ml) and CaCl_2_ (1 mM) were added to microsomes, and the mixture was incubated for 12 min at room temperature, before inactivation with 4 mM EGTA. Following nuclease treatment, microsomes were isolated at 160,000 ***g*** through a sucrose cushion (0.5 M sucrose, 50 mM HEPES pH 7.5 and 100 mM KCL), resuspended in DPM buffer (0.25 M sucrose, 25 mM HEPES pH 7.5 and 50 mM KCL) and stored at −80°C.

HT1080 (CCL-121) cell lines were purchased from the American Type Culture Collection (ATCC) and were recently authenticated using the Eurofins cell line authentication service. The ERp57 KO, ERdj5 KO and ERp57/ERdj5 KO cell lines were created as described previously (van Lith et al., 2021). All cell lines were tested for mycoplasma contamination and found to be negative. Cells were cultured in DMEM (Gibco) supplemented with 2 mM glutamine (Gibco), 100 U/ml penicillin (Sigma), 100 µg/ml streptomycin (Sigma) and 10% fetal calf serum (Sigma). SP cells were prepared using digitonin as described previously (Wilson et al., 1995). SP cells were nuclease treated (10 μg/ml micrococcal nuclease, 1 mM CaCl_2_) for 12 min at room temperature before inactivation of nuclease with EGTA (4 mM). Nuclease-treated SP cells were pelleted and resuspended in KHM buffer for immediate use in translation reactions at ∼10^5^ cells per 25 μl translation reaction.

### Cell-free translation

The Flexi® Rabbit Reticulocyte Lysate System (Promega), was either supplemented with 5 mM DTT (Melford) to make reducing lysate, 2.5 mM G6P (Sigma) to make balanced lysate or left untreated to make oxidising lysate. The following components were added to the lysate to make the translation mix: amino acids except methionine (20 μM), KCl (40 mM), EasyTag^TM^ EXPRESS^35^S Protein Labeling Mix (PerkinElmer Life Sciences, 1 μl per 25 μl reaction), RNA template, and SP cells or microsomes. For translations containing castanospermine (Sigma), this was also added to a final concentration of 1 mM.

Following assembly of components, translation reactions were incubated at 30°C for either 10 min (disintegrin translations) or 30 min (LDLr or β1-integrin translations). For G6P recovery experiments, following the initial translation in oxidising lysate, the reactions were supplemented with G6P (2.5 mM) and incubated for a further 10 min or 30 min. Samples without G6P addition were performed in parallel for comparison. On completion, all samples were treated with 20 mM NEM (Sigma), to block further redox changes during downstream processing. Samples containing SP cells were isolated through centrifugation (16,000 ***g*** for 30 s) and washed twice using KHM buffer (20 mM HEPES pH 7.2, 110 mM KOAc, 2 mM MgOAc). Samples containing microsomes were isolated through centrifugation (16,000 ***g***, 5 min) and washed twice using 20 mM HEPES pH 7.5.

### Immunoisolation and ConA precipitation

Either immunoprecipitation (IP) buffer [50 mM Tris-HCl pH 7.5, 1% (v/v) Triton X-100, 150 mM NaCl, 2 mM EDTA, 0.5 mM PMSF, and 0.02% (w/v) sodium azide] was used for both incubation and wash steps or beads buffer (10 mM HEPES pH 7.4, 200 mM NaCl, 2.5 mM MgCl_2_, 2 mM CaCl_2_ ,0.1% Triton X-100, 0.5 mM PMSF, 0.1% BSA) was used for the incubation steps and wash buffer (50 mM Tris-HCl pH 8.6. 150 mM NaCl, 1% Triton X-100, 0.5% SDS) was used for the wash steps. Either Protein A–Sepharose (PAS; Generon) or Protein G–Sepharose (PGS; Generon) or a mixture of both (PAS/PGS) were used as indicated.

IP buffer with PGS was used for experiments shown in Figs 2 and 3B. Beads buffer and wash buffer with PAS/PGS was used for experiments shown in Fig. 3C,D, Fig. 4C, right panels, Fig. 5C–E and Fig. 6A. IP buffer with PAS was used for experiments in Fig. 4C, left panels, and Fig. 5B. In all cases the membrane or cell pellets from the translation reactions were resuspended in 100–500 µl buffer with 30 µl of 10% beads (v/v) for 1 h at 4°C. The samples were centrifuged at 2000 ***g*** for 1 min to remove beads and then the supernatant was incubated with fresh beads and the indicated antibody at 4°C overnight. The antibodies used were anti-V5 antibody (Invitrogen, cat. no. 46-0705) used at 1:10,000, the neo-epitope antibody (Robinson et al., 2020) used at 1:500, the 9EG7 antibody (BD Pharmigen cat. no. 553715) used at 1:100 and the LDLR C7 antibody (Santa Cruz Biotechnology, cat. no. sc-18823) used at 1:40. Following the overnight incubation, samples were centrifuged at 2000 ***g*** for 1 min to pellet beads, the supernatant was discarded and fresh buffer was added to wash the beads. The wash steps were repeated a further two times before a final wash with 20 mM HEPES buffer, pH 7.5.

For ConA isolation, the same incubation and wash procedure was followed as described for immunoisolations but using ConA incubation buffer [50 mM Tris-HCl pH 7.5, 1% (v/v) Triton X-100, 150 mM NaCl, 1 mM MgCl_2_, 1 mM MnCl_2_ and 1 mM CaCl_2_, 0.5 mM PMSF, and 0.02% (w/v) sodium azide] in the incubation steps and ConA wash buffer (50 mM Tris-HCl pH 8.6, 150 mM NaCl, 1% Triton X-100, 0.5% SDS, 1 mM MgCl_2_, 1 mM MnCl_2_ and 1 mM CaCl_2_) in the wash steps. The preclear step took place using PAS/PGS beads and the incubation step with concanavalin A–Sepharose 4B (Sigma).

### EndoH treatment

Immunoisolated samples that required EndoH treatment were boiled in 0.5% SDS for 5 mins then allowed to cool. Sodium acetate was added (50 mM pH 6.0) and 1 µl of EndoH (NEB, P0702L). Samples were incubated for 1 h at 37°C.

### SDS-PAGE

Beads containing immune isolated material were resuspended in non-reducing SDS-PAGE buffer (120 mM Tris-HCl pH 6.8, 4% (w/v) SDS, 20% (v/v) glycerol, 0.04% (w/v) Bromophenol Blue) and boiled for 5 mins. Samples run under reducing conditions were treated with 5 mM DTT and boiled for a further 5 mins. All samples were treated with 50 mM NEM to block free thiol groups. LDLr and β1-integrin samples were typically run on 7.5% polyacrylamide gels, disintegrin samples were typically run on 15% polyacrylamide gels. Following electrophoresis, gels were fixed in 10% acetic acid (v/v) / 10% methanol (v/v), dried and exposed to a phosphorimager plate for analysis on a FLA-7000 bioimager (Fujifilm).

### Reagent availability

All requests for reagents should be directed to the corresponding author.
