# Supplementary material for: Distinct role of ERp57 and ERdj5 as a disulfide isomerase and reductase during ER protein folding
Source: J Cell Sci. 2023 Jan 19;136(2):jcs260656. doi: 10.1242/jcs.260656 (PMC10022741; doi:10.1242/jcs.260656)
Supplement: Supplementary file 1 [file joces-136-260656_review_history.pdf]

Distinct role of ERp57 and ERdj5 as a disulfide isomerase and reductase during ER protein folding

Philip John Robinson, Marie Anne Pringle, Bethany Fleming and Neil John Bulleid
DOI: 10.1242/jcs.260656

Editor: Jennifer Lippincott-Schwartz

Review timeline

Original submission:	22 September 2022
Editorial decision:	31 October 2022
First revision received:	30 November 2022
Accepted:	13 December 2022

Original submission

First decision letter

MS ID#: JOCES/2022/260656

MS TITLE: Distinct role of ERp57 and ERdj5 as disulfide isomerase and reductase during ER protein folding.

AUTHORS: Philip John Robinson, Marie Anne Pringle, Bethany Fleming, and Neil J Bulleid
ARTICLE TYPE: Research Article

We have now reached a decision on the above manuscript.

To see the reviewers' reports and a copy of this decision letter, please go to: https://submit-jcs.biologists.org and click on the 'Manuscripts with Decisions' queue in the Author Area. (Corresponding author only has access to reviews.)

As you will see, the reviewers raise a number of substantial criticisms that prevent me from accepting the paper at this stage. They suggest, however, that a revised version might prove acceptable, if you can address their concerns. If you think that you can deal satisfactorily with the criticisms on revision, I would be pleased to see a revised manuscript. We would then return it to the reviewers.

Please ensure that you clearly highlight all changes made in the revised manuscript. Please avoid using 'Tracked changes' in Word files as these are lost in PDF conversion.

I should be grateful if you would also provide a point-by-point response detailing how you have dealt with the points raised by the reviewers in the 'Response to Reviewers' box. Please attend to all of the reviewers' comments. If you do not agree with any of their criticisms or suggestions please explain clearly why this is so.

Reviewer 1

Advance summary and potential significance to field

In their paper entitled "Distinct role of ERp57 and ERdj5 as disulfide isomerase and reductase during ER protein folding" Robinson et al. address important questions in the cell biology of the ER:

© 2023. Published by The Company of Biologists under the terms of the Creative Commons Attribution License (https://creativecommons.org/licenses/by/4.0/).

why do we have so many different PDIs, do they have distinct functions? The authors mostly use semi-permeabilized cells to address these questions as they allow to specifically modulate the redox environment. Focusing on three (stalled) protein substrates, the authors find that ERp57 is mainly involved in isomerizing disulfide bonds in glycoproteins, whereas ERdj5 has more reductive functions. For ERdj5, this is consistent with and extends previous studies on this protein. Taken together, this study provides relevant new insights into oxidative protein folding in the ER by revealing, for two ER PDI family members, subtrate-specific effects on correct disulfide bond formation.

Comments for the author

The following points should be addressed prior to publication:

1. On page 6, the authors claim that “Each intermediate translated in reducing lysate ran as a prominent band”. This reviewer does not see this “prominent band”, not drastically more than for the bal/ox conditions. This raises the question why this would be the case, if indeed conditions are reducing. In the same experiment, why would the protein migrate faster under ox conditions, where predominantly short range disulfide bonds of adjacent cysteines may form?

2. In Fig. 3Bii, correct structure formation is also observed under ox conditions, whereas before it says that only in balanced lysate this occurred. This needs to be reconciled. Also, in Fig 3Bii it would be helpful to indicate where the reduced protein migrates.

3. Fig. 4C shows in lane 1 a species at approx. 26kDa that is not mentioned

4. For ERdj5 and BiP, substrate binding studies are available. Does this help in rationalizing the effects the authors observe on different proteins?

Reviewer 2

Advance summary and potential significance to field

In this clearly and concisely written manuscript, Robinson et al investigate a crucial aspect of oxidative protein folding, that is the relative roles of oxidation isomerization and reduction in the biogenesis of complex proteins.

To do so, they exploit an in vitro translation system to translocation into microsomes or semi-permeabilized cells devoid of ERp57 and ERdj5. The former is thought to act as an isomerase, and the latter as a reductase.

Three client proteins with different requirements are investigated: an integrin, ADAM10 and LDLR. Full length or translation arrest constructs are used to prime the system and conformational antibodies and high resolution gel electrophoreses to dissect some key steps in the processes of disulfide bond formation.

Comments for the author

The experiments are carefully performed, and most key controls are in place to support the conclusions reached.

The results presented confirm and extend our knowledge on a fundamental problem of molecular cell biology.

The authors may wish to consider the following points before the manuscript goes into print.

Owing to the abundance of other oxidoreductases, it is quite surprising that the loss of Erp57 is not at least partly compensated by Erdj5.

© 2023. Published by The Company of Biologists under the terms of the Creative Commons Attribution License (https://creativecommons.org/licenses/by/4.0/).

Rescue experiments with different enzymes might strengthen the authors' case.
A castanospermine experiment might tell whether calreticulin and calnexin are needed in this system.

Reviewer 3

Advance summary and potential significance to field

Robinson et al. reported the requirement of ER resident oxidoreductases for the disulfide formation of nascent polypeptides for the correct folding during the co-translational translocation into the ER. Authors focuses on two enzymes ERp57 known as oxidase/isomerase and ERdj5 known as reductase for this reaction.

For this experiment, authors newly developed the in vitro translation system with SP cells or microsomes with or without the addition of G6P. Authors also adopted the stop codon-less mRNA for examining the co-translational disulfide formation as well as isomerization. This system enables us to analyse oxidation and isomerization distinctively, which might be worth to be reported.

However, the conclusion described here is just the confirmation of previously reported pieces of the enzymatic characterization of ERp57 and ERdj5, and novelty obtained from the results shown here is not high enough. In addition three substrates were used and the results on the effect of ERp57 and ERdj5 were described for each one, but the effects of these two enzymes on different substrates cannot be extrapolated for general substrates containing multiple disulfide bonds, which might weaken the validity of this manuscript to be published in this journal.

Comments for the author

1. Detailed explanations for the experimental procedures are repeatedly described in the Result section, which should be described in the Materials and Methods.

2. Fig. 4C. In the presence of G6P, the mature band is hardly seen either for Wt or for double KO cells. For the conclusion described at the top of page 10, this should be improved.

3. Information provided from Fig. 7 is too poor to be shown as the conclusive figure.

First revision

Author response to reviewers' comments

Response to reviewers

We thank the reviewers for their time and effort in commenting on this manuscript. Their suggestions have been well received and we hope to have addressed all their concerns. We respond to the individual comments below. For ease of reference, we have highlighted all the text changes to the manuscript in red.

Reviewer 1

We note the very positive comments made by the reviewer and in particular that the work "provides relevant new insights into oxidative protein folding in the ER".

Points to be addressed:

> *1. On page 6, the authors claim that "Each intermediate translated in reducing lysate ran as a prominent band". This reviewer does not see this "prominent band", not drastically more*

© 2023. Published by The Company of Biologists under the terms of the Creative Commons Attribution License (https://creativecommons.org/licenses/by/4.0/).

than for the bal/ox conditions. This raises the question why this would be the case, if indeed conditions are reducing

Our intention was to describe the reduced band as being the most prominent within the lane rather than between lanes. To avoid confusion, we have removed the word “prominent” and replaced it with “slow migrating”.

We commonly observe small difference in expression levels between samples, but it is the change in gel mobility, which is a reliable indicator of disulfide formation and the shift between samples is clear.

1. In the same experiment, why would the protein migrate faster under ox conditions, where predominantly short-range disulfide bonds of adjacent cysteines may form?

The gel migration expected for different disulfide bond configurations in the same proteins is difficult to predict and can depend on such factors as transient folding states and substrate interactions. In our experience non-native, long range disulfide bonds commonly form in disulfide rich substrates under oxidising conditions. We have added a sentence to the results emphasise that this is occurring and included a reference where the same phenomenon is observed for LDLR.

2. In Fig. 3Bii, correct structure formation is also observed under ox conditions, whereas before it says that only in balanced lysate this occurred. This needs to be reconciled. Also, in Fig 3Bii it would be helpful to indicate where the reduced protein migrates.

The difference in correct folding under -G6Pi conditions between Fig 2 and Fig 3 can be explained by the longer translation time (30 min in 2C vs 60 min in 3Bii) and the use of SP cells instead of microsomes. Folding is known to be more efficient in SP cells than microsomes and the extra translation time may also assist native folding. The improvement in folding efficiency is therefore not unexpected. We have included this explanation in the text.

We moved the gel in Fig 3Bi so that it aligns with Fig 3 Bii and added a dotted line to indicate the migration of the reduced protein.

3. Fig. 4C shows in lane 1 a species at approx. 26kDa that is not mentioned

This band was also observed in our previous paper (Robinson et al. 2020). Its slow migration indicates an interchain disulfide that forms under ox conditions. We have added a short explanation in the text to cover this point.

4. For ERdj5 and BiP, substrate binding studies are available. Does this help in rationalizing the effects the authors observe on different proteins?

It is well established that BiP recognises aliphatic residues that are typically buried in proteins, while more recently it has been shown that ERdj5 specifically binds aggregation prone sequences. We do not think this explains the effects we see, because ß-1-integrin interactions with ERdj5 have been detected. We have included extra sentences in the discussion to highlight this with relevant references added.

Reviewer 2

We note again the very positive comments made by the reviewer, and in particular that “the results presented confirm and extend our knowledge on a fundamental problem of molecular cell biology”.

Points to be addressed:

1. *Owing to the abundance of other oxidoreductases, it is quite surprising that the loss of Erp57 is not at least partly compensated by Erdj5. Rescue experiments with different enzymes might strengthen the authors’ case.*

© 2023. Published by The Company of Biologists under the terms of the Creative Commons Attribution License (https://creativecommons.org/licenses/by/4.0/).

The lack of compensation by ERdj5 and other oxidoreductases for the loss of ERp57 during ß-1-integrin folding is striking, especially with the knowledge that ERdj5 can compensate during the folding of LDLR. The suggestion to perform rescue experiments is a good one but would require a lot of work to generate knock-in cell lines. We hope to do this in future as part of further work to decipher the ER reducing pathway. However, we think that the castanospermine experiment that the reviewer also suggested (described below) helps strengthen the case. Castanospermine blocks access to ERp57 by preventing entry into the calnexin cycle. The fact that castanospermine has the same influence on folding as is seen with the ERp57 knockout supports our findings that ß-1-integrin is strictly ERp57 dependent.

2. *A castanospermine experiment might tell whether calreticulin and calnexin are needed in this system.*

We have performed a castanospermine experiment as suggested using the ß-1-integrin construct. This has been included as a new panel in Figure 3 and is described in the results section.

Reviewer 3

We thank the reviewer for their comments. Although we agree that some of our findings confirm previous results, our study provides new information in terms of the dependence of ERp57 and ERdj5 on the cytosolic reductive pathway. Furthermore, new insights are gained into the activity of the enzymes with folding substrates. These include the role of ERp57 in LDLR folding and how ERdj5 can compensate in its absence if a robust reducing pathway is active. We therefore believe that our study provides an important step forward in understanding the ER-reductive pathway. We are conscience that our findings cannot be extrapolated directly to other proteins in the ER, but we think that our results have highlighted how different substrates have different enzymatic requirements for disulfide isomerisation during folding, which will have relevance for the folding of many secretory proteins and will therefore help to direct future studies.

We address below the specific points raised

1. *Detailed explanations for the experimental procedures are repeatedly described in the Result section, which should be described in the Materials and Methods.*

Our aim was to make the results section easier to understand by describing the experiments in detail, however we agree that this was too detailed in some parts and better suited to the methods. We have edited the results section to remove repeated details, such as NEM treatments and IP procedures and either deleted them, moved descriptions to the methods or integrated them elsewhere in the results section.

2. *Fig. 4C. In the presence of G6P, the mature band is hardly seen either for Wt or for double KO cells. For the conclusion described at the top of page 10, this should be improved.*

We think that the mature band is clear in the original data but may have become less clear during processing/ compression. To improve this, we have adjusted the brightness/contrast of the gel and added some extra annotations so that mature band is more prominent and clear to see.

3. *Information provided from Fig. 7 is too poor to be shown as the conclusive figure.*

We would prefer to leave this figure in the paper as we think it provides a useful visual summary of our findings and will help the reader to understand the work. But we will remove it if the editor thinks it is necessary to do so.

© 2023. Published by The Company of Biologists under the terms of the Creative Commons Attribution License (https://creativecommons.org/licenses/by/4.0/).

Second decision letter

MS ID#: JOCES/2022/260656

MS TITLE: Distinct role of ERp57 and ERdj5 as disulfide isomerase and reductase during ER protein folding.

AUTHORS: Philip John Robinson, Marie Anne Pringle, Bethany Fleming, and Neil J Bulleid
ARTICLE TYPE: Research Article

I am happy to tell you that your manuscript has been accepted for publication in Journal of Cell Science, pending standard ethics checks.

© 2023. Published by The Company of Biologists under the terms of the Creative Commons Attribution License (https://creativecommons.org/licenses/by/4.0/).
